# Retrospective Analysis of Zolbetuximab‐Associated Hypoalbuminemia in Claudin 18.2‐Positive Advanced Gastric Cancer

**DOI:** 10.1002/cnr2.70709

**Published:** 2026-10-14

**Authors:** Taro Mizuno, Yukiya Narita, Taiju Ando, Yasunobu Ishizuka, Sho Umegaki, Kazunori Honda, Toshiki Masuishi, Hiroya Taniguchi, Shigenori Kadowaki, Masashi Ando, Masahiro Tajika, Kei Muro

**Affiliations:** ^1^ Department of Clinical Oncology Aichi Cancer Center Hospital Nagoya Japan; ^2^ Center for Cancer Genomics and Advanced Therapeutics, Aichi Cancer Center Hospital Nagoya Japan; ^3^ Department of Endoscopy Aichi Cancer Center Hospital Nagoya Japan

**Keywords:** claudin 18.2, gastric cancer, hypoalbuminemia, zolbetuximab

## Abstract

**Background:**

Hypoalbuminemia has been recognized as a characteristic adverse event associated with zolbetuximab plus chemotherapy in the treatment of claudin 18.2‐positive advanced gastric cancer. However, its incidence, clinical predictors, and management in real‐world settings remain unclear.

**Aims:**

We aimed to characterize the course of hypoalbuminemia during zolbetuximab‐based therapy and identify factors associated with its severity and recovery.

**Methods and Results:**

We retrospectively analyzed patients with advanced gastric cancer treated with zolbetuximab plus chemotherapy at a single center, tracking serial serum albumin levels. Severe hypoalbuminemia was defined as a nadir albumin < 2.0 g/dL and/or a ≥ 1.0 g/dL decrease from baseline. Associations between severe hypoalbuminemia and clinicopathological factors were assessed, along with its management strategies. A total of 39 patients were included in the analysis. Hypoalbuminemia occurred in all patients, and 20 (51.3%) developed severe hypoalbuminemia. Prior total gastrectomy was associated with a lower incidence of severe hypoalbuminemia (0% vs. 58.8%, *p* = 0.02), whereas persistent nausea and/or vomiting beyond the zolbetuximab infusion during the first cycle was associated with a higher risk (63.0% vs. 25.0%, *p* = 0.04). Recovery of serum albumin levels was observed in 18 patients (46.2%) and occurred more frequently among those who temporarily interrupted zolbetuximab plus chemotherapy or zolbetuximab alone (66.7% vs. 28.6%, *p* = 0.03).

**Conclusion:**

Severe hypoalbuminemia was common in patients receiving zolbetuximab plus chemotherapy in real‐world practice. The absence of prior total gastrectomy and the presence of persistent post‐infusion nausea and/or vomiting may help identify patients at risk. The observed association between temporary treatment interruption and albumin recovery may provide insights into the management of hypoalbuminemia.

## 1 Introduction

Gastric cancer is the fifth most common cancer and one of the leading causes of cancer‐related mortality worldwide [1]. Although the prognosis for patients with advanced gastric cancer (AGC) remains poor, the development of molecularly targeted agents and immune checkpoint inhibitors, in combination with chemotherapy, has led to incremental improvements in survival [2, 3, 4, 5, 6].

Zolbetuximab, a chimeric monoclonal antibody targeting claudin‐18 isoform 2 (CLDN18.2), has been approved in combination with platinum‐ and fluoropyrimidine‐based chemotherapy as a first‐line treatment for patients with CLDN18.2‐positive AGC. This agent has a distinctive safety profile, characterized particularly by a high incidence of nausea and vomiting that often occurs early during treatment, prompting the development of practical prevention and management strategies in clinical practice [7, 8]. Additionally, hypoalbuminemia and the resulting peripheral edema have increasingly been recognized as characteristic adverse events associated with zolbetuximab‐based therapy [8, 9, 10]. Because these events may lead to nutritional deterioration and treatment interruption, they have important implications for treatment feasibility in routine clinical practice. However, despite their potential clinical impact, the incidence, temporal profile, and optimal management of zolbetuximab‐associated hypoalbuminemia remain insufficiently characterized, particularly in real‐world settings outside clinical trials. In pivotal phase III trials, the reported incidence of hypoalbuminemia was relatively modest. In an integrated analysis of the SPOTLIGHT and GLOW trials, hypoalbuminemia was reported in 19.3% of patients for all grades and in only 3.8% for grade ≥ 3 events, suggesting that its clinical significance may have been underestimated in controlled trial settings [11].

Accordingly, in this retrospective study, we aimed to characterize the temporal changes in serum albumin levels associated with zolbetuximab‐based therapy, identify clinically relevant risk factors, and explore practical management strategies applicable to real‐world clinical practice.

## 2 Methods

### 2.1 Patients and Treatment

This single‐center retrospective study was conducted at the Aichi Cancer Center Hospital. Patients with AGC who initiated zolbetuximab plus chemotherapy between July 2024 and August 2025 and received at least two cycles of treatment were included. Patients who received only one treatment cycle were excluded because, based on the serum albumin assessment schedule described below, no post‐baseline pre‐cycle measurement was available to evaluate longitudinal changes. Eligibility for zolbetuximab required immunohistochemical confirmation of human epidermal growth factor receptor 2 (HER2)‐negative, CLDN18.2‐positive gastric or esophagogastric junction adenocarcinoma. The infusion protocol for zolbetuximab and the accompanying premedication regimen were predefined at our institution [8]. All patients received standard antiemetic premedication consisting of a serotonin 5‐HT3 receptor antagonist (granisetron), a Neurokinin‐1 receptor antagonist (fosnetupitant), and dexamethasone before zolbetuximab infusion. Additionally, all patients received a potassium‐competitive acid blocker (vonoprazan) and rebamipide for gastric mucosal protection during treatment courses. Treatment modulation was at the discretion of the treating oncologist, following routine clinical practice.

This study was approved by the Institutional Review Board of Aichi Cancer Center Hospital (IRB reference number: 2025‐0‐660) and was conducted in accordance with the ethical principles of the Declaration of Helsinki. The requirement for written informed consent was waived due to the retrospective, non‐interventional nature of the study, and an opt‐out option was provided on the institutional website.

### 2.2 Evaluation

Baseline characteristics, adverse events, and clinical outcomes were collected from medical records. Adverse events were graded according to the Common Terminology Criteria for Adverse Events (CTCAE), version 5.0 [12].

Serum albumin levels were measured at baseline and before each subsequent treatment cycle, and the incidence and characteristics of hypoalbuminemia were evaluated. To investigate clinicopathological factors associated with severe hypoalbuminemia, it was defined as a minimum serum albumin level < 2.0 g/dL and/or a decrease of ≥ 1.0 g/dL from baseline to the nadir value during treatment. The cutoff of < 2.0 g/dL corresponds to CTCAE grade 3 hypoalbuminemia [12], whereas the additional criterion of a ≥ 1.0 g/dL decrease from baseline was adopted in accordance with the definition used by Shimozaki et al. [10]. This criterion was intended to capture clinically meaningful albumin deterioration during therapy that may not be fully reflected by CTCAE grading based on absolute serum albumin levels, particularly in patients with relatively high baseline levels.

The association between severe hypoalbuminemia and treatment efficacy was also examined using tumor response, ascites response, progression‐free survival (PFS), and overall survival (OS). Tumor response in patients with measurable lesions was assessed according to the Response Evaluation Criteria in Solid Tumors (RECIST), version 1.1 [13]. Ascites response in patients with baseline ascites was evaluated as follows: complete response (CR), disappearance of ascites; partial response (PR), improvement by at least one severity level; stable disease (SD), no CR, PR, or progression; progressive disease (PD), worsening by at least one severity level; and not evaluable (NE), absence of computed tomography (CT) imaging. Ascites severity was classified as massive (extending throughout the entire abdominal cavity), moderate (not meeting the criteria for mild or massive), mild (localized to either the upper or lower abdomen), or absent (undetectable on CT). The objective response rate (ORR) was defined as the proportion of patients with measurable lesions who achieved CR or PR as their best overall response. The ascites response rate was defined as the proportion of patients with baseline ascites who achieved CR or PR as their best ascites response. PFS was defined as the time from initiation of zolbetuximab‐containing chemotherapy to documented disease progression or death from any cause, whichever occurred first. OS was defined as the time from treatment initiation to death from any cause.

### 2.3 Statistical Analysis

The ORR and ascites response rate were presented with two‐sided 95% exact confidence intervals (CIs) calculated using the Clopper–Pearson method. PFS and OS were estimated using the Kaplan–Meier method and compared between patients with and without severe hypoalbuminemia using the log‐rank test. Hazard ratios (HRs) and 95% CIs were estimated using a Cox proportional hazards model. Associations of clinicopathological factors with severe hypoalbuminemia were assessed using the chi‐square test or Fisher's exact test, as appropriate. Univariable logistic regression with Firth's penalized likelihood was performed to estimate odds ratios and 95% CIs for these associations. A *p* value < 0.05 was considered statistically significant. All statistical analyses were performed using EZR (Jichi Medical University, Tochigi, Japan), a graphical user interface for R (R Foundation for Statistical Computing, Vienna, Austria) [14].

### 2.4 Use of Artificial Intelligence Tools

During the preparation of this manuscript, ChatGPT (OpenAI) was used to assist with English‐language editing and refinement of the organization and presentation of the text. All AI‐assisted text was reviewed and revised by the authors to ensure its accuracy. The AI tool was not used for data collection, data analysis, or determination of the scientific interpretation. The authors take full responsibility for the content of the manuscript.

## 3 Results

### 3.1 Patient Characteristics

Of the 45 patients who received zolbetuximab, six were excluded for the following reasons: treatment with zolbetuximab monotherapy (*n* = 3) and receipt of only one treatment cycle (*n* = 3). Consequently, 39 patients were included in the analysis (Figure S1). Baseline characteristics are summarized in Table 1. The median age was 61 years (range, 31–81), and 20 patients (51.3%) were male. Most patients had a gastric primary tumor (89.7%), and 13 patients (33.3%) had undergone prior gastrectomy (total gastrectomy in five patients, distal gastrectomy in seven, and esophagectomy in one). The peritoneum was the most common metastatic site (84.6%), and ascites was present in 25 patients (64.1%). Histologically, 31 patients (79.5%) had diffuse‐type adenocarcinoma. The median serum albumin level was 3.7 g/dL (range, 2.3–4.6 g/dL). Thirty‐one patients (79.5%) received zolbetuximab plus chemotherapy as first‐line therapy, whereas eight patients (20.5%) received it as second‐line or later therapy. Backbone chemotherapy regimens were 5‐fluorouracil–based in 22 patients (56.4%) and S‐1‐ or capecitabine‐based in 17 patients (43.6%).

**TABLE 1:** Patient characteristics.

Characteristic	Categories	*n* = 39
Age, years	Median (range)	61 (31–81)
Sex	Male	20 (51.3)
	Female	19 (48.7)
ECOG PS	0	30 (76.9)
	1	9 (23.1)
Primary site	Stomach	35 (89.7)
	EGJ	4 (10.3)
Borrmann type	Type 4	22 (56.4)
	Non‐type 4	17 (43.6)
Primary resection	Yes	13 (33.3)
	Total gastrectomy	5 (12.8)
	Distal gastrectomy	7 (17.9)
	Others^a^	1 (2.6)
	No	26 (66.7)
Inadequate oral intake	Yes	6 (15.4)
	No	33 (84.6)
Location of metastases	Peritoneum	33 (84.6)
	Lymph node	14 (35.9)
	Liver	1 (2.6)
Ascites	Present	25 (64.1)
	Massive	9 (23.1)
	Moderate	3 (7.7)
	Mild	13 (33.3)
	Absent	14 (35.9)
Organs with metastases	0–1	24 (61.5)
	≥ 2	15 (38.5)
Histological type	Diffuse	31 (79.5)
	Intestinal, others	8 (20.5)
Serum albumin, g/dL	Median (range)	3.7 (2.3–4.6)
NLR	Median (range)	2.68 (0.80–13.3)
Line of therapy	First‐line	31 (79.5)
	≥ Second‐line	8 (20.5)
Backbone chemotherapy	5‐FU‐based	22 (56.4)
	S‐1‐or capecitabin‐based	17 (43.6)

*Note:* Data are presented as *n* (%) unless otherwise indicated.

Abbreviations: 5‐FU, 5‐fluorouracil; ECOG PS, Eastern Cooperative Oncology Group performance status; EGJ, esophagogastric junction; NLR, neutrophil‐to‐lymphocyte ratio.

^a^ Esophagectomy.

### 3.2 Serum Albumin Kinetics and Peripheral Edema

Within a median follow‐up of 175 days, all patients developed hypoalbuminemia of any grade; 24 patients (61.5%) experienced grade 2 and seven patients (17.9%) experienced grade 3 hypoalbuminemia (Table 2). Serum albumin levels declined in all but one patient, and the median time to reach the minimum level during treatment was 34 days (range, 14–340 days). Peripheral edema of any grade occurred in 18 patients (46.1%), and diuretics were administered to 16 patients (41.0%). Notably, among patients who experienced recovery, serum albumin levels gradually improved after reaching the nadir, particularly following temporary interruption of treatment (Figure 1).

**TABLE 2:** Incidence and characteristics of hypoalbuminemia and peripheral edema during treatment.

Characteristics	Categories	*n* = 39
Hypoalbuminemia	Any grade	39 (100.0)
	Grade 1	8 (20.5)
	Grade 2	24 (61.5)
	Grade 3	7 (17.9)
Decrease from Alb_base_		38 (97.4)
Time to Alb_min_, days	Median (range)	34 (14–340)
Peripheral edema	Any grade	18 (46.1%)
	Diuretics use	16 (41.0%)

*Note:* Data are presented as *n* (%) unless otherwise indicated.

Abbreviations: Alb_base_, baseline albumin level; Alb_min_, minimum albumin level.

**FIGURE 1:**
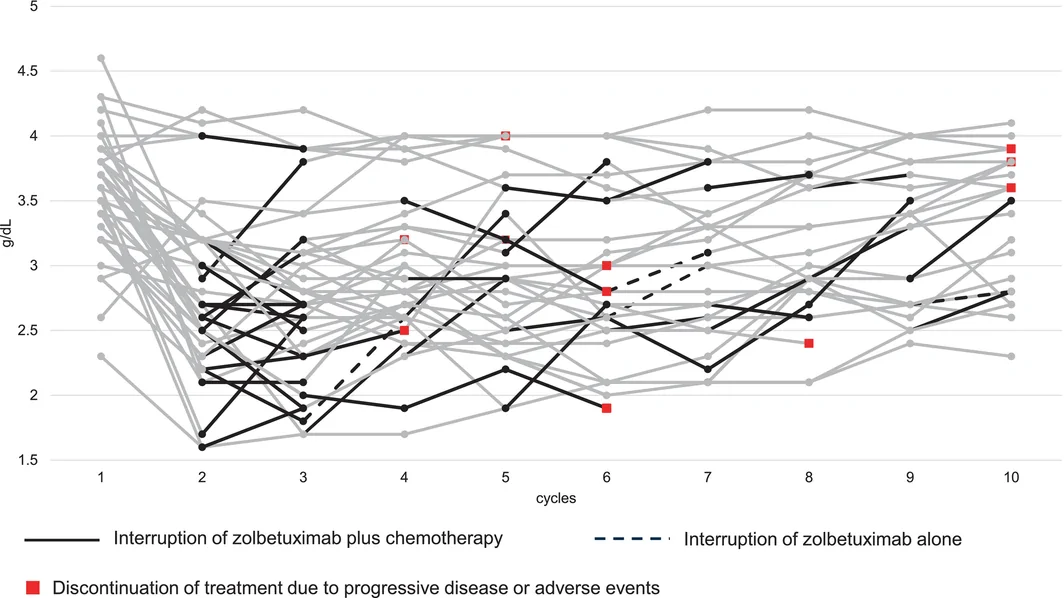
Changes in serum albumin levels through treatment cycle 10.

### 3.3 Association Between Severe Hypoalbuminemia and Clinicopathological Factors

Twenty patients (51.3%) developed severe hypoalbuminemia, as previously defined. Patients with nausea, vomiting, or both lasting from the completion of the first cycle to the initiation of second cycle of zolbetuximab infusion had a significantly higher incidence of severe hypoalbuminemia than those without these symptoms (63.0% vs. 25.0%, *p* = 0.04). In contrast, no cases of severe hypoalbuminemia were observed among patients with prior total gastrectomy (0% vs. 58.8%, *p* = 0.02) (Table 3). Nausea, vomiting, or abdominal pain requiring temporary interruption of zolbetuximab infusion during the first cycle (85.7% vs. 43.8%, *p* = 0.09) and anorexia during the first cycle (58.1% vs. 25.0%, *p* = 0.13) tended to be associated with a higher incidence of severe hypoalbuminemia, although these differences were not statistically significant. Other factors, including the primary tumor site, histological subtype, backbone chemotherapy regimen, and line of therapy, were not associated with the development of severe hypoalbuminemia (Table S1). Univariable Firth's penalized logistic regression analysis also showed similar findings (Figure S2).

**TABLE 3:** Association between severe hypoalbuminemia and clinicopathological factors.

Variables	Severe hypoalbuminemia		*p*
	Yes	No	
Total	20 (51.3)	19 (48.7)	
Total gastrectomy			
Yes	0 (0.0)	5 (100.0)	0.02
No	20 (58.8)	14 (41.2)	
Persistent N/V after Zol infusion during C1			
Yes	17 (63.0)	10 (37.0)	0.04
No	3 (25.0)	9 (75.0)	
N/V/A requiring interruption of Zol infusion at C1			
Yes	6 (85.7)	1 (14.3)	0.09
No	14 (43.8)	18 (56.3)	
Anorexia during C1			
Yes	18 (58.1)	13 (41.9)	0.13
No	2 (25.0)	6 (75.0)	

*Note:* Data are presented as *n* (%).

Abbreviations: C1, cycle 1; N/V/A, nausea/vomiting/abdominal pain; Zol, zolbetuximab.

### 3.4 Outcomes After Reaching the Minimum Albumin Level

Recovery of serum albumin levels from the nadir to baseline was observed in 18 patients (46.2%), with a median time to recovery of 64.5 days (range, 14–177 days). No patients received albumin supplementation. Patients who temporarily interrupted treatment for any reason after reaching the nadir were significantly more likely to experience recovery of serum albumin levels from the nadir to baseline compared with those who continued treatment (66.7% vs. 28.6%, *p* = 0.03) (Table 4). Treatment was interrupted in 18 patients—both zolbetuximab and chemotherapy in 16 patients and zolbetuximab alone in two patients—with a median interruption duration of 14 days (range, 7–43 days). Because only a small number of patients received anamorelin for cancer cachexia or tranexamic acid for protein‐losing enteropathy, analyses of their therapeutic efficacy were not performed.

**TABLE 4:** Association between temporary treatment interruption and recovery of serum albumin.

Variable	Recovery from Alb_min_ to Alb_base_		*p*
	Yes	No	
Total	18 (46.2)	21 (53.8)	
Temporary interruption			
Yes	12 (66.7)	6 (33.3)	0.03
No	6 (28.6)	15 (71.4)	

*Note:* Data are presented as *n* (%).

Abbreviations: Alb_base_, baseline albumin level; Alb_min_, minimum albumin level.

### 3.5 Association Between Severe Hypoalbuminemia and Treatment Efficacy

Among the 10 patients with at least one measurable lesion according to the RECIST version 1.1, ORR was 20.0% (95% CI, 0.5–71.6) in patients without severe hypoalbuminemia and 60.0% (95% CI, 14.7–94.7) in those with severe hypoalbuminemia (*p* = 0.52). Among the 25 patients with baseline ascites, the ascites response rate was 50.0% (95% CI, 18.7–81.3) in patients without severe hypoalbuminemia and 40.0% (95% CI, 16.3–67.7) in those with severe hypoalbuminemia (*p* = 0.70) (Figure S3).

The median follow‐up period for the survival analyses was 7.1 months. PFS and OS were analyzed separately according to the presence or absence of severe hypoalbuminemia and by line of therapy (Figure S4). Among patients receiving first‐line therapy, the median PFS was 11.1 months (95% CI, 2.3–not reached [NR]) in patients without severe hypoalbuminemia and 8.0 months (95% CI, 5.6–NR) in those with severe hypoalbuminemia (HR, 0.85; 95% CI, 0.24–2.95; *p* = 0.80). The corresponding median OS values were 12.3 months (95% CI, 12.3–NR) and 14.0 months (95% CI, NR–NR), respectively (HR, 0.27; 95% CI, 0.03–2.61; *p* = 0.22). Similarly, among patients receiving second‐ or later‐line therapy, neither PFS nor OS differed significantly according to severe hypoalbuminemia (*p* = 0.84 and *p* = 0.50, respectively).

## 4 Discussion

This real‐world study provides several clinically important insights into zolbetuximab‐associated hypoalbuminemia. First, the incidence and severity of hypoalbuminemia observed in routine clinical practice were substantially higher than those reported in pivotal clinical trials. Second, prior total gastrectomy and persistent post‐infusion nausea, vomiting, or both during the first treatment cycle emerged as clinically relevant factors associated with the development of severe hypoalbuminemia. Third, nearly half of the patients experienced recovery of serum albumin levels, and temporary treatment interruption was associated with this recovery, raising the possibility that treatment‐related albumin deterioration may be partially reversible.

In pivotal phase III trials, clinically significant hypoalbuminemia was considered uncommon; however, in our real‐world cohort, hypoalbuminemia occurred in all patients, with more than half developing severe hypoalbuminemia [11]. This discrepancy between controlled clinical trials and routine practice suggests that zolbetuximab‐associated hypoalbuminemia may be driven by on‐target effects on normal gastric mucosa that are not fully captured under trial conditions. Consistent with a previous report by Shimozaki et al. [10], a history of total gastrectomy was strongly associated with the absence of severe hypoalbuminemia, supporting the mechanistic hypothesis that the presence of CLDN18.2‐expressing normal gastric mucosa plays a central role in susceptibility to zolbetuximab‐induced albumin loss. Protein‐losing enteropathy (PLE) is a syndrome characterized by gastrointestinal loss of plasma proteins, presenting as otherwise unexplained hypoalbuminemia and edema and supported by objective evidence of protein leakage, such as radiotracer albumin scintigraphy. An emerging report of zolbetuximab‐induced PLE, characterized by diffuse gastric mucosal redness and edema, mucous cell detachment and fundic gland disruption on biopsy, and direct visualization of albumin leakage on Tc‐99m albumin scintigraphy, further supports this mucosa‐centered mechanism and closely mirrors the clinical features observed in our study [15].

Although gastrointestinal toxicities during zolbetuximab infusion in the first cycle, such as nausea, vomiting, and abdominal pain, require careful attention as potential markers of acute mucosal irritation, they were not significantly associated with severe hypoalbuminemia in our study. Notably, unlike previous reports that primarily focused on infusion‐limited events [10], our evaluation also included gastrointestinal symptoms persisting beyond the infusion period. Importantly, our study newly identified that nausea and vomiting persisting beyond the infusion during the first cycle were significantly associated with severe hypoalbuminemia, suggesting that ongoing post‐infusion mucosal injury may be a more clinically informative marker than infusion‐limited events.

Another strength of this study lies in the detailed characterization of albumin kinetics after reaching the nadir. Nearly half of the patients experienced recovery of serum albumin, and temporary treatment interruption was significantly associated with albumin recovery. This finding suggests that zolbetuximab‐induced mucosal injury may be at least partially reversible when the drug is withheld. However, the present analysis could not evaluate whether albumin recovery following treatment interruption translated into clinical benefits. Particularly, as most patients with edema received diuretics, whether improvement in edema was attributable to treatment interruption or diuretic therapy was difficult to determine. Moreover, most patients interrupted both zolbetuximab and chemotherapy rather than zolbetuximab alone, potentially compromising treatment efficacy. Therefore, further studies are needed to investigate the optimal management of hypoalbuminemia.

Regarding treatment efficacy, no clear differences between patients with and without severe hypoalbuminemia were observed in ORR, ascites response rate, PFS, or OS. However, the effect estimates were imprecise, as reflected by the wide 95% CIs, and the survival data remained immature because of the limited number of events and relatively short follow‐up period. In addition, the PFS and OS analyses may have been subject to time‐dependent bias because patients were grouped according to severe hypoalbuminemia that developed after treatment initiation. These findings should therefore be regarded as exploratory and should not be interpreted as demonstrating the prognostic significance of severe hypoalbuminemia or a causal relationship with survival outcomes.

An association between diffuse gastric mucosal inflammation and hypoalbuminemia has been reported by Yamamoto et al. [9] In the present study, all patients were managed according to our institutional administration protocol, which includes routine use of acid‐suppressive agents and gastric mucosal protectants, as previously described [8]. Nevertheless, hypoalbuminemia occurred in all patients, suggesting that these measures alone may be insufficient to prevent its development. Furthermore, given the potential reversibility of this condition, excessive or non‐protocol‐based prophylactic medication may be unnecessary, and careful monitoring with timely treatment modification may represent a more appropriate management approach.

Several limitations should be acknowledged. First, the small sample size and retrospective, single‐institution design may have limited statistical power and introduced residual confounding. Second, hypoalbuminemia may also arise from the underlying disease, and disease progression during the clinical course may have affected serum albumin levels. To reduce the potential influence of hypoalbuminemia associated with later disease progression, we performed a supplementary analysis restricted to the first 12 weeks after treatment initiation, which showed trends generally consistent with those observed over the entire treatment period (Table S2). Nevertheless, the contribution of disease‐related factors could not be fully excluded, and the causal relationship between zolbetuximab‐containing treatment and hypoalbuminemia should therefore be interpreted cautiously. Third, the decrease of ≥ 1.0 g/dL used as an additional criterion for severe hypoalbuminemia was a study‐specific threshold that has not been validated, and the optimal cutoff remains uncertain. Fourth, routine endoscopic assessment was not performed, preventing direct correlation between clinical hypoalbuminemia and gastric mucosal injury.

In conclusion, severe hypoalbuminemia was common during zolbetuximab plus chemotherapy in routine clinical practice. The absence of prior total gastrectomy and the presence of persistent post‐infusion nausea, vomiting, or both may help identify patients at increased risk of profound albumin decline, supporting close monitoring of serum albumin levels. Although temporary treatment interruption was associated with albumin recovery, its clinical benefit could not be established in this small, retrospective study. Further validation is required to determine the optimal management of hypoalbuminemia without compromising treatment efficacy.

## Author Contributions

**Taro Mizuno:** conceptualization, methodology, data curation, investigation, formal analysis, visualization, writing – original draft. **Yukiya Narita:** conceptualization, methodology, data curation, investigation, validation, project administration, writing – review and editing. **Taiju Ando:** writing – review and editing. **Yasunobu Ishizuka:** writing – review and editing. **Sho Umegaki:** writing – review and editing. **Kazunori Honda:** writing – review and editing. **Toshiki Masuishi:** writing – review and editing. **Hiroya Taniguchi:** writing – review and editing. **Shigenori Kadowaki:** writing – review and editing. **Masashi Ando:** writing – review and editing. **Masahiro Tajika:** writing – review and editing. **Kei Muro:** writing – review and editing, project administration, supervision.

## Funding

The authors have nothing to report.

## Consent

The requirement for individualized informed consent was waived due to the retrospective nature of the study. A committee‐approved information disclosure document was published on the hospital's website.

## Conflicts of Interest

The authors declare the following financial interests/personal relationships which may be considered potential competing interests:

Taro Mizuno: honoraria for lectures from Astellas and Merck BioPharma.

Yukiya Narita: research funding to my institution from Chugai, MSD, Amgen, ONO Pharmaceutical, Astellas, Sanofi, Taiho, Eisai, Daiichi Sankyo, Novartis, and Pfizer; honoraria for lectures, presentations, and speaker bureaus from Taiho, Eli Lilly, Daiichi Sankyo, ONO Pharmaceutical, Bristol‐Myers Squibb, and Astellas; and participation on the Advisory Board of Daiichi Sankyo and Astellas.

Kazunori Honda: honoraria for lectures, presentations, and speaker bureaus from Kyowa Kirin, Eisai, Chugai, AstraZeneca, and Celltrion Healthcare Japan.

Toshiki Masuishi: research funding to my institution from MSD, Daiichi Sankyo, ONO Pharmaceutical, Novartis, Amgen, Syneos Health Clinical, Boehringer Ingelheim, Pfizer, Cimic Shift Zero, Eli Lilly, AbbVie, and Merck BioPharma; consulting fees from Nippon Kayaku; honoraria for lectures, presentations, and speaker bureaus from Takeda, Chugai, Merck BioPharma, Taiho, Bayer, Eli Lilly, Yakult Honsha, Sanofi, Daiichi Sankyo, ONO Pharmaceutical, Bristol‐Myers Squibb, Nippon Kayaku, MSD, Takata, Guardant Health, and Amgen.

Hiroya Taniguchi: research funding to my institution from Takeda and Daiichi Sankyo; honoraria for lectures, presentations, and speaker bureaus from Takeda and Eli Lilly.

Shigenori Kadowaki: research funding to my institution from Ono Pharmaceutical, MSD, Janssen Pharmaceutical, Eli Lilly, Daiichi Sankyo, AstraZeneca, Taiho, Nobel Pharmaceutical, Bayer, Chugai, and Abbvie; honoraria for lectures, presentations, and speaker bureaus from Ono Pharmaceutical, MSD, Merck KGaA, Eli Lilly, Bayer, Novartis, Taiho, Daiichi Sankyo, Bristol‐Myers Squibb, Chugai, and Eisai.

Masashi Ando: honoraria for lectures, presentations, and speaker bureaus from Eisai, Ono Pharmaceutical, and Taiho.

Masahiro Tajika: honoraria for educational events from Bristol‐Myers Squibb and EA Pharma.

Kei Muro: research funding to my institution from Amgen, Taiho, PRA Health Sciences, Chugai, Eli Lilly, and AstraZeneca; consulting fees from Amgen, AstraZeneca, Ono Pharmaceutical, Astellas, and Chugai; honoraria for lectures from Ono Pharmaceutical, Bristol‐Myers Squibb, Astellas, MSD, Takeda, and Daiichi Sankyo.

## Supporting information


**Figure S1:** Study flow diagram.
**Figure S2:** Univariable firth's penalized logistic regression analysis of factors associated with severe hypoalbuminemia.
**Figure S3:** (A) Best overall response in patients with measurable lesions. (B) Maximum percentage change in tumor burden from baseline in patients with measurable lesions. (C) Best ascites response in patients with baseline ascites.
**Figure S4:** (A) PFS and (B) OS in patients receiving first‐line therapy. (C) PFS and (D) OS in patients receiving second‐ or later‐line therapy.

**Table S1:** Association of additional clinicopathological factors with severe hypoalbuminemia.
**Table S2:** Association between severe hypoalbuminemia and clinicopathological factors within 12 weeks after treatment initiation.

## Data Availability

The datasets used and analyzed during the current study are available from the corresponding author on reasonable request.
