# Supplementary material for: Retrospective Analysis of Zolbetuximab‐Associated Hypoalbuminemia in Claudin 18.2‐Positive Advanced Gastric Cancer
Source: Cancer Rep (Hoboken). 2026 Oct 14;9(10):e70709. doi: 10.1002/cnr2.70709 (PMC13667797; doi:10.1002/cnr2.70709)
Supplement: Supplementary file 1 [file CNR2-9-e70709-s001.docx]

**Table S1. Association of additional clinicopathological factors with severe hypoalbuminemia**

| Variable | Severe hypoalbuminemia | | *P* value |
| --- | --- | --- | --- |
|  | Yes | No |  |
| **Total** | 20 (51.3) | 19 (48.7) |  |
| **Age** |  | | |
| ≥65 | 9 (60.0) | 6 (40.0) | 0.51 |
| <65 | 11 (45.8) | 13 (54.2) |  |
| **Sex** |  |  |  |
| Male | 9 (45.0) | 11 (55.0) | 0.53 |
| Female | 11 (57.9) | 8 (42.1) |  |
| **ECOG PS** |  |  |  |
| 1 | 3 (33.3) | 6 (66.7) | 0.27 |
| 0 | 17 (56.7) | 13 (43.3) |  |
| **Primary site** |  |  |  |
| Stomach | 19 (54.3) | 16 (45.7) | 0.34 |
| EGJ | 1 (25.0) | 3 (75.0) |  |
| **Borrmann type** |  |  |  |
| Type 4 | 10 (45.5) | 12 (54.5) | 0.52 |
| Non-type 4 | 10 (58.8) | 7 (41.2) |  |
| **Primary resection** |  |  |  |
| Yes | 6 (46.2) | 7 (53.8) | 0.74 |
| No | 14 (53.8) | 12 (46.2) |  |
| **Inadequate oral intake** |  |  |  |
| Yes | 3 (50.0) | 3 (50.0) | 1.00 |
| No | 17 (51.5) | 16 (48.5) |  |
| **Ascites** |  |  |  |
| Present | 15 (60.0) | 10 (40.0) | 0.19 |
| Absent | 5 (35.7) | 9 (64.3) |  |
| **Organs with metastases** |  |  |  |
| ≥2 | 8 (53.3) | 7 (46.7) | 1.00 |
| 0–1 | 12 (50.0) | 12 (50.0) |  |
| **Histology** |  |  |  |
| Diffuse | 17 (54.8) | 14 (45.2) | 0.45 |
| Intestinal, others | 3 (37.5) | 5 (62.5) |  |
| **NLR** |  |  |  |
| ≥Median | 9 (47.4) | 10 (52.6) | 0.75 |
| <Median | 11 (55.0) | 9 (45.0) |  |
| **Backbone chemotherapy** |  |  |  |
| 5-FU-based | 13 (59.1) | 9 (40.9) | 0.34 |
| S-1- or Cape-based | 7 (41.2) | 10 (58.8) |  |
| **Line of therapy** |  |  |  |
| ≥2nd | 4 (50.0) | 4 (50.0) | 1.00 |
| 1st | 16 (51.6) | 15 (48.4) |  |

Data are presented as n (%).

Abbreviations: ECOG PS, Eastern Cooperative Oncology Group performance

status; EGJ, esophagogastric junction; NLR, neutrophil-to-lymphocyte ratio; 5-FU, 5-fluorouracil.

**Table S2. Association between severe hypoalbuminemia and clinicopathological factors within 12 weeks after treatment initiation**

| Variable | Severe hypoalbuminemia | | *P* value |
| --- | --- | --- | --- |
|  | Yes | No |  |
| **Total** | 19 (48.7) | 20 (51.3) |  |
| **Age** |  | | |
| ≥65 | 8 (53.3) | 7 (46.7) | 0.75 |
| <65 | 11 (45.8) | 13 (54.2) |  |
| **Sex** |  |  |  |
| Male | 9 (45.0) | 11 (55.0) | 0.75 |
| Female | 10 (52.6) | 9 (47.4) |  |
| **ECOG PS** |  |  |  |
| 1 | 3 (33.3) | 6 (66.7) | 0.45 |
| 0 | 16 (53.3) | 14 (46.7) |  |
| **Primary site** |  |  |  |
| Stomach | 18 (51.4) | 17 (48.6) | 0.61 |
| EGJ | 1 (25.0) | 3 (75.0) |  |
| **Borrmann type** |  |  |  |
| Type 4 | 10 (45.5) | 12 (54.5) | 0.75 |
| Non-type 4 | 9 (52.9) | 8 (47.1) |  |
| **Primary resection** |  |  |  |
| Yes | 5 (38.5) | 8 (61.5) | 0.50 |
| No | 14 (53.8) | 12 (46.2) |  |
| **Total gastrectomy** |  |  |  |
| Yes | 0 (0.0) | 5 (100.0) | 0.05* |
| No | 19 (55.9) | 15 (44.1) |  |
| **Inadequate oral intake** |  |  |  |
| Yes | 3 (50.0) | 3 (50.0) | 1.00 |
| No | 16 (48.5) | 17 (51.5) |  |
| **Ascites** |  |  |  |
| Present | 14 (56.0) | 11 (44.0) | 0.32 |
| Absent | 5 (35.7) | 9 (64.3) |  |
| **Organs with metastases** |  |  |  |
| ≥2 | 8 (53.3) | 7 (46.7) | 0.75 |
| 0–1 | 11 (45.8) | 13 (54.2) |  |
| **Histology** |  |  |  |
| Diffuse | 17 (54.8) | 14 (45.2) | 0.24 |
| Intestinal, others | 2 (25.0) | 6 (75.0) |  |
| **NLR** |  |  |  |
| ≥Median | 8 (42.1) | 11 (57.9) | 0.53 |
| <Median | 11 (55.0) | 9 (45.0) |  |
| **Backbone chemotherapy** |  |  |  |
| 5-FU-based | 12 (54.5) | 10 (45.5) | 0.52 |
| S-1- or Cape-based | 7 (41.2) | 10 (58.8) |  |
| **Line of therapy** |  |  |  |
| ≥2nd | 3 (37.5) | 5 (62.5) | 0.69 |
| 1st | 16 (51.6) | 15 (48.4) |  |
| **Persistent N/V after Zol infusion during C1** |  |  |  |
| Yes | 16 (59.3) | 11 (40.7) | 0.08 |
| No | 3 (25.0) | 9 (75.0) |  |
| **N/V/A requiring interruption of Zol infusion at C1** |  |  |  |
| Yes | 5 (71.4) | 2 (28.6) | 0.24 |
| No | 14 (43.8) | 18 (56.3) |  |
| **Anorexia during C1** |  |  |  |
| Yes | 17 (54.8) | 14 (45.2) | 0.24 |
| No | 2 (25.0) | 6 (75.0) |  |

Data are presented as n (%).

**P* <0.05. Significance was determined using unrounded *P* values.

Abbreviations: C1, cycle 1; ECOG PS, Eastern Cooperative Oncology Group performance status; EGJ, esophagogastric junction; NLR, neutrophil-to-lymphocyte ratio; N/V/A, nausea/vomiting/abdominal pain; Zol; zolbetuximab; 5-FU, 5-fluorouracil.
