# Supplementary material for: Retrospective Analysis of Zolbetuximab‐Associated Hypoalbuminemia in Claudin 18.2‐Positive Advanced Gastric Cancer
Source: Cancer Rep (Hoboken). 2026 Oct 14;9(10):e70709. doi: 10.1002/cnr2.70709 (PMC13667797; doi:10.1002/cnr2.70709)
Supplement: Supplementary file 2 [file CNR2-9-e70709-s002.zip › cnr270709-sup-0001-Supinfo2.docx]

**Figure S1. Study flow diagram.**

**Figure S2. Univariable Firth’s penalized logistic regression analysis of factors associated with severe hypoalbuminemia.**

**P* <0.05.

Abbreviations: ECOG PS, Eastern Cooperative Oncology Group performance status; EGJ, esophagogastric junction; NLR, neutrophil-to-lymphocyte ratio; N/V/A, nausea/vomiting/abdominal pain; Ref, reference; Zol, zolbetuximab; 5-FU, 5-fluorouracil.

**Figure S3. (A) Best overall response in patients with measurable lesions. (B) Maximum percentage change in tumor burden from baseline in patients with measurable lesions. (C) Best ascites response in patients with baseline ascites.**

†Patients treated in the second-line or later setting.

Abbreviations: CI, confidence interval; CR, complete response; NE, not evaluable; NS, not significant; ORR, objective response rate; PD, progressive disease; PR, partial response; SD, stable disease.

**Figure S4. (A) PFS and (B) OS in patients receiving first-line therapy. (C) PFS and (D) OS in patients receiving second- or later-line therapy.**

Abbreviations: CI, confidence interval; HR, hazard ratio; NR, not reached; Ref, reference.
